# Functionalised thermally induced phase separation (TIPS) microparticles enabled for “click” chemistry†
†Electronic supplementary information (ESI) available. See DOI: 10.1039/d0ob00106f


**DOI:** 10.1039/d0ob00106f

**Published:** 2020-03-02

**Authors:** João C. F. Nogueira, Ketevan Paliashvili, Alexandra Bradford, Francesco Di Maggio, Daniel A. Richards, Richard M. Day, Vijay Chudasama

**Affiliations:** a UCL Chemistry Department , University College London , Gower Street , London , WC1E 6BT , UK . Email: v.chudasama@ucl.ac.uk; b Centre for Precision Healthcare , UCL Division of Medicine , University College London , Gower Street , London , WC1E 6BT , UK . Email: r.m.day@ucl.ac.uk; c The Discoveries Centre for Regenerative and Precision Medicine , University College London , Gower Street , London , WC1E 6BT , UK

## Abstract

Due to their homogeneity, tuneable properties, low cost and ease of manufacture, thermally induced phase separation (TIPS) polymeric microparticles are emerging as an exciting class of injectable device for the treatment of damaged tissue or complex diseases, such as cancer. However, relatively little work has explored enhancing surface functionalisation of this system. Herein, we present the functionalisation of TIPS microparticles with both small molecules and an antibody fragment of Herceptin™, *via* a heterobifunctional pyridazinedione linker capable of participating in SPAAC “click” chemistry, and compare it to the traditional method of preparing active-targeted microparticle systems, that is, physisorption of antibodies to the microparticle surface. Antigen-binding assays demonstrated that functionalisation of microparticles with Herceptin Fab, *via* a pyridazinedione linker, provided an enhanced avidity to HER2+ when compared to traditional physisorption methods.

## Abstract 2

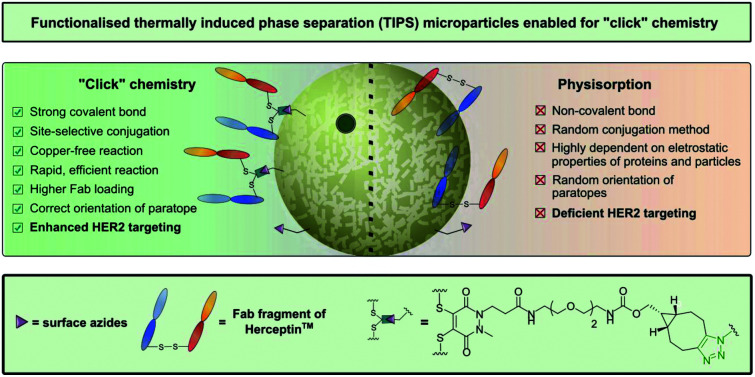
In this study we describe a novel platform for generating functionalised TIPS microparticles for “click” conjugation to various active compounds.

## Introduction

Microparticle-based platforms are well-established in the market due to their emergent use for research and development purposes (*e.g.* Dynabeads)^1^ and for a plethora of therapeutic and diagnostic applications.^2^–^4^ Poly(lactic-*co*-glycolic acid) (PLGA) microparticles are amongst the most widely used due to their important biocompatible, biodegradable and non-toxic properties.^5^ With various applications in drug delivery, microparticles composed of PLGA allow release of encapsulated materials in a controlled manner, whilst protecting the materials against undesirable degradative reactions, therefore, permitting release of therapeutic drugs at controlled rates for the desired period of time.^6^,^7^ Furthermore, PLGA microparticle systems are attracting more attention in the field of regenerative medicine as potential injectable scaffolds, since they can be delivered minimally invasively *in situ* and have the ability to conform to the shape of the implant site.^8^ Although there are several methods for producing PLGA microparticles (*e.g.* solvent-emulsion evaporation, spray-drying or static mixing)^9^ a novel methodology allowing rapid formation of monodisperse highly-porous particles involves the use of thermally induced phase separation (TIPS).^7^,^10^,^11^ The TIPS method not only permits tailoring of microparticle size (from nano- to micro-scale in diameter), porosity and pore morphology but also allows inclusion of active ingredients such as small molecules and protein based therapies into the polymer matrix, as well providing a delivery vehicle for advanced therapies.^8^,^11^–^14^

**Fig. 1:**
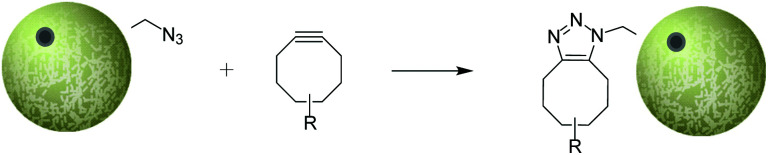
Azide-functionalised TIPS microparticles undergoing SPAAC “click” modification. R: small molecule, protein.

A further beneficial attribute of PLGA polymeric microparticles is their compatibility with a wide range of materials, including synthetic polymers^15^ and biological materials such as antibodies, enabling targeting of specific disease biomarkers.^16^ To date, most of the loading of active ingredients such as antibodies or small molecules into micro/nanoparticle systems has been achieved by either blending the compounds into the polymer solution during the fabrication process^11^,^12^,^16^ or using post-fabrication random conjugation methods (electrostatic or covalent) to append the two entities, *e.g.* physisorption or covalently attaching antibodies (*via* random surface lysine residue modification) to particles *via* carbodiimide chemistry.^14^,^17^,^18^ However, in the case of loading microparticles with antibody, the aforementioned procedures greatly limit antigen-binding and overall target avidity, as well making it difficult to control aggregation. We have recently demonstrated the importance of controlled chemical “click” ligation of proteins for successful nanoconjugate performance, particularly in the context of target affinity.^19^,^20^ However, this work has been limited to a specific polymersome with no application to any other particular delivery vehicles. The current study explored the feasibility of applying “click” chemistry to TIPS microparticles, to attach both small molecules and a site-selectively modified clinically-relevant Fab of Herceptin™. To ensure modularity, we created a platform that is amenable to commonly employed and well-tolerated copper (Cu)-free “click” chemistry, namely strain-promoted azide–alkyne cycloaddition (SPAAC) chemistry (Fig. 1). Additionally, we report the design of a modified ELISA assay, specific for this system, that allows newly-formed microparticle–antibody conjugates (generated *via* SPAAC) to be tested for HER2 binding affinity.

## Results and discussion

### Synthesis of PLGA and PLGA-N_3_ TIPS microparticles

**Fig. 2:**
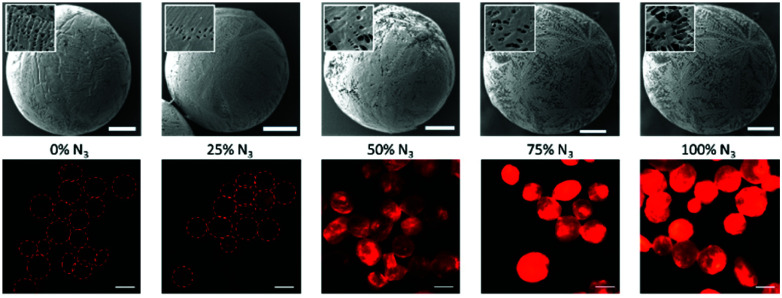
TIPS microparticles with 0%, 25%, 50%, 75% and 100% surface azide (top) and fluorescence microscopy of each of the microparticles with a TAMRA strained alkyne derivative. Scale bars for SEM images = 100 μm and for immunofluorescence images = 300 μm.

Our study began with the synthesis of a series of TIPS microparticles that contained differing ratios of PLGA : PLGA-N_3_ (ranging from 0% to 100% PLGA-N_3_). An advantageous feature of the process used to fabricate TIPS microparticles is that it does not require aqueous washing stages to remove the solvent from the polymer, unlike in most commonly utilised methods (*e.g.* in the formation of PCL–azide microparticles).^21^ Furthermore, the TIPS microparticle processing technique provides greater control over porosity of the microparticles compared with conventional solvent-emulsion evaporation techniques used to manufacture microparticles. After synthesis, the range of composite TIPS microparticles investigated that contained the different ratios of PLGA : PLGA-N_3_ did not exhibit any marked changes to their surface structural features (Fig. 2). All of the microparticles exhibited hierarchically structured surface porosity characteristic of the TIPS manufacturing process, with surface pores frequently organised into chevron-like patterns.

### Functionalisation of PLGA-N_3_ microparticles with fluorophore

Detection of azide-capped PLGA on the surface of TIPS microparticles capable of providing a stable triazole linkage through SPAAC chemistry was performed using fluorescence microscopy with an azadibenzocyclooctyne-tetramethyl rhodamine (TAMRA) derivative. The dibenzocyclooctyne successfully reacted with the azide group of the functionalized PLGA, resulting in increased fluorescence signal that was approximately proportional to the quantity of azide-capped PLGA in the TIPS microparticles (Fig. 2). Based on the uniformly strong fluorescence labelling with the TAMRA derivative in TIPS microparticles composed of 75% PLGA-N_3_, this composition of the composite microparticles was used in subsequent experiments in the current proof-of-concept study.

### Functionalisation of PLGA-N_3_ microparticles with Fab antibody fragment

Having demonstrated that the azide bearing TIPS microparticles could be successfully generated and reacted *via* SPAAC chemistry, we set out to appraise whether we could translate this strategy for the attachment of a clinically relevant protein in a controlled manner, *i.e.* by site-selective modification. The Fab of monoclonal antibody Herceptin™ was chosen as the protein platform due to its clinical relevance as an approved therapeutic against HER2+ cancers.^17^ The decision to utilise the Fab of a full antibody was driven by a number of factors: (i) the substantial literature inferring antibody fragments/small protein scaffold provide multiple benefits over full antibodies; (ii) Fabs contains a single solvent accessible disulfide bond (distal from the binding site) to ensure homogeneous modification by our previously reported disulfide re-binding technology;^20^,^22^ (iii) Fabs can be readily expressed and/or obtained from native full antibody scaffolds *via* simple enzymatic digestion procedures.^17^

**Fig. 3:**
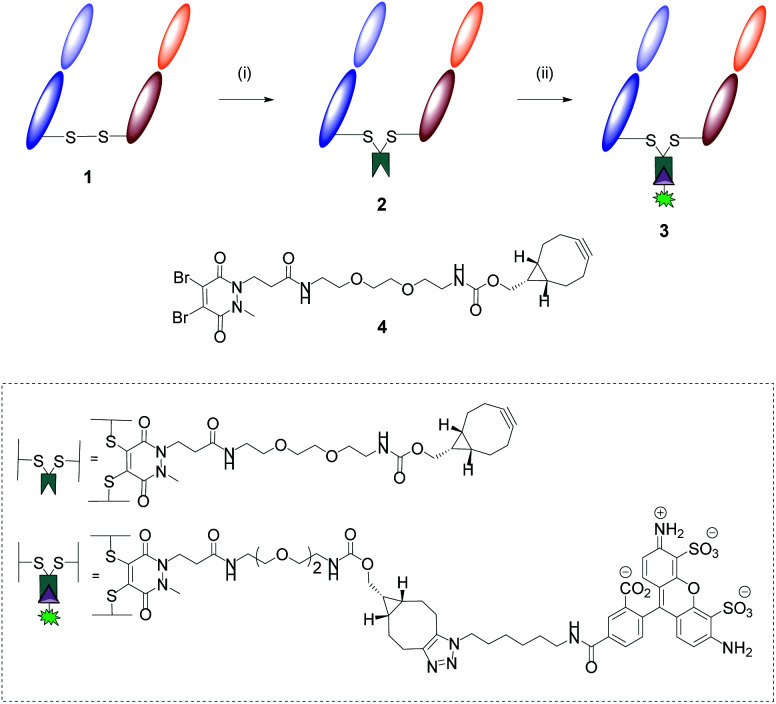
Modification of Herceptin™ Fab with reagent **4** and subsequent “click” test reaction. Reagents and conditions: (i) Reagent **4**, TCEP·HCl, borate buffer pH 8.0 (5 mM EDTA), 21 °C, 16 h; (ii) Alexafluor®-488-N_3_, phosphate buffer (pH 7.4), 21 °C, 2 h.

This part of the study began with the formation of Herceptin™ Fab from full antibody Herceptin™ by enzymatic digestion (pepsin followed by papain, further details provided in the ESI†). Site-selective modification of Herceptin™ Fab with strained alkyne-pyridazinedione reagent **4** was achieved according to previously reported protocols (Fig. 3).^23^ Subsequently, reactivity of strained alkyne was confirmed *via* SPAAC “click” reaction with Alexafluor®-488-N_3_, yielding fluorescent conjugate **3** (Fig. 3).

The aforementioned conjugates were successfully characterised *via* multiple analytic methods (LCMS, UV/Vis and SDS-PAGE, details provided in the ESI†) and showed no aggregation or fragmentation.

**Fig. 4:**
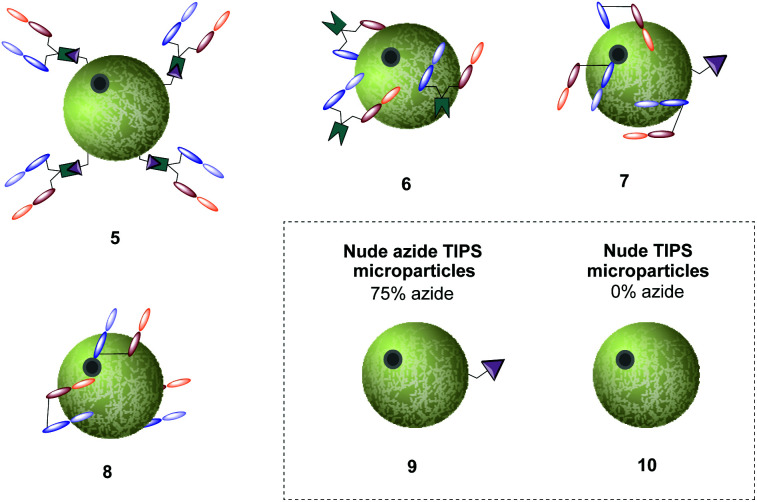
Representative images of generated Fab-TIPS microparticles and nude-TIPS microparticle controls (75% and 0% azide loading).

With the availability of Herceptin™ Fab strained alkyne conjugate **2**, we sought to explore how well it could be conjugated to 75% azide bearing TIPS microparticles **9**. Since TIPS microparticles **9** and **10** are insoluble in aqueous buffer, the addition of detergent Tween 80 to phosphate buffered saline (PBS) was fundamental for particle immersion into solution. Subsequently, “click” reaction between azide-bearing microparticles and Herceptin Fab strained alkyne conjugate **2** was performed, generating the antibody–microparticle “clicked” conjugate, hereafter referred to as conjugate **5**. As the standard method for attaching proteins to the surface of microparticles is physisorption, we sought to use this as our main control in this study. Thus, Herceptin Fab was incubated with 75% and 0% azide bearing TIPS microparticles, generating physisorbed antibody–microparticle conjugates **7** and **8**, respectively. To dismiss the possibility that addition of Herceptin Fab strained alkyne conjugate **2** was increasing the amount of physiosorbed antibody fragments on the surface (*i.e.* when compared to addition of native Herceptin Fab) rather than react *via* SPAAC “click” to the microparticles, Herceptin™ Fab strained alkyne conjugate **2** was also incubated with 0% azide TIPS (control **6**). Finally, 75% azide bearing TIPS microparticles **9** and nude TIPS microparticles **10** were also included as controls (Fig. 4).

### Anti-human IgG (Fab)-peroxidase binding assay

Having successfully developed conjugate **5** and controls, we next explored detection of Fab in the conjugate using anti-human IgG (Fab)-peroxidase to quantify Fab loading of each conjugate. For this purpose, we developed an ELISA assay that consisted of analysing the microparticles in Costar® Spin-X® centrifuge tube filters, which simplifies the washing process and avoids further microparticle handling (further details in the ESI†).

**Fig. 5:**
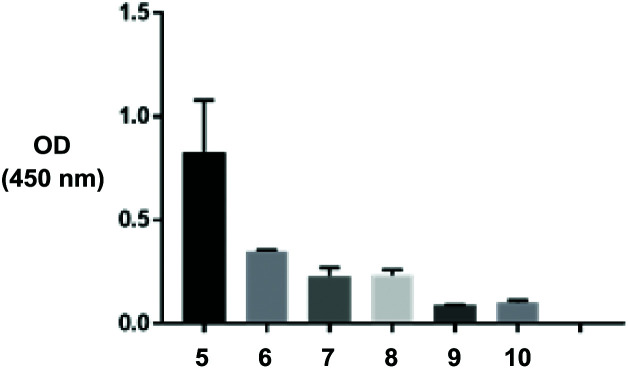
Modified ELISA demonstrating Fab loading and availability on various TIPS microparticles. Experiment was run in duplicate.

After thorough optimisation of several parameters (*e.g.* number of washes between steps, amount of Fab and detection antibody added to microparticles, addition of surfactants) the assay demonstrated increased binding of anti-human IgG (Fab specific)-peroxidase to particles prepared *via* SPAAC click chemistry (conjugate **5**) *versus* physisorption (conjugates **6**, **7**) and associated controls (Fig. 5). This indicates a greater surface loading of Fab fragments on the particle surface was achieved using the designed click chemistry. These results were expected – attachment of relatively small proteins *via* a non-specific interaction such as physisorption is frequently inferior to covalent chemistries, unless the surface is highly charged.

### HER2+ binding assay

**Fig. 6:**
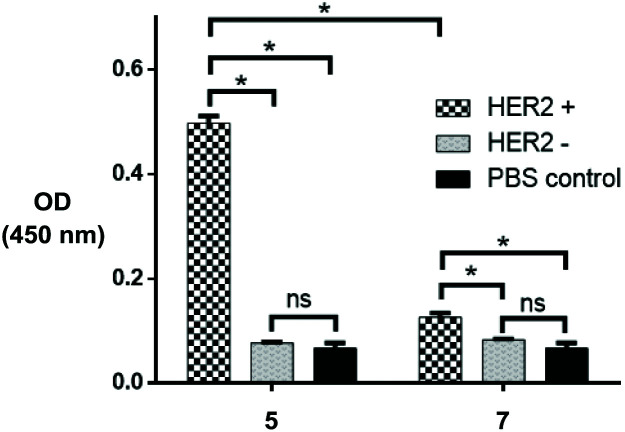
ELISA data demonstrating successful targeting of click modified TIPS microparticles (**5**) and particles generated *via* physisorption (**7**) towards HER2. Experiment was run in triplicate. Statistical significance (*, *p* = 0.01) was determined *via* two-way ANOVA, using Tukey's multiple comparison test.

Having determined successful conjugation of the Fab fragment to the particles, we sought to investigate binding of the HER2 antigen to the azide bearing TIPS microparticles. For this, we took on the optimised constructs and performed a sandwich ELISA assay, where biotin-conjugated HER2 and HRP-conjugated streptavidin were used as capture and detection antibodies respectively (details in the ESI†). HER2-negative controls and PBS controls with nude microparticles were also performed in parallel (Fig. 6). The subsequent results showed superior HER2 binding to conjugate **5** when compared with remaining controls and, relevantly, with HER2-positive physisorbed control **7**, demonstrating that a more controlled covalent approach allows for greater HER2 targeting. Additionally, results demonstrate that the HER2-negative controls are not considerably different to the PBS control, demonstrating that there is minimal non-specific binding between the particles and HER2.

## Conclusions

In this study we describe a novel platform for generating functionalised TIPS microparticles for conjugation of active compounds. For this, we used a modular method for covalently attaching Fab fragments onto the surface of TIPS microparticles, using a pyridazinedione linker capable of participating in SPAAC “click” chemistry. The azide bearing TIPS microparticle conjugates showed enhanced avidity for the target when compared with the traditional physisorbed control.

## Conflicts of interest

There are no conflicts to declare.

## Supplementary Material

Supplementary information
