# Supplementary material for: Functionalised thermally induced phase separation (TIPS) microparticles enabled for “click” chemistry
Source: Org Biomol Chem. 2020 Mar 2;18(12):2215–8. doi: 10.1039/d0ob00106f (PMC7362741; doi:10.1039/d0ob00106f)
Supplement: Supplementary file 1 [file OB-018-D0OB00106F-s001.pdf]

Electronic Supplementary Material (ESI) for Organic & Biomolecular Chemistry.
This journal is © The Royal Society of Chemistry 2020

Electronic Supplementary Information

Functionalized thermally induced phase separation (TIPS) microparticles enabled for "*click*" chemistry

João Carlos Nogueira[a], Ketevan Paliashvili[b], Alexandra Bradford[b], Francesco Di Maggio[b], Daniel A. Richards[a], Richard M. Day[b,c*], Vijay Chudasama[a*]

[a] UCL Chemistry Department, University College London, Gower Street, London, WC1E 6BT, UK

[b] Centre for Precision Healthcare, UCL Division of Medicine, University College London, Gower Street, London, WC1E 6BT, UK

[c] The Discoveries Centre for Regenerative and Precision Medicine, University College London, Gower Street, London, WC1E 6BT, UK

General Experimental

All reagents and starting materials were obtained from chemical suppliers, unless specifically stated otherwise, and were used as received. Reactions were monitored by thin layer chromatography using pre-coated SIL G/UV 254 plates purchased from VWR. Flash chromatography was carried out automatically using a BioTage Isolera with KP-Snap columns. NMR spectra were recorded using a Bruker AC600 spectrometer (600 MHz). Chemical shifts (δ) are given in ppm units relative to the solvent reference and coupling constants (*J*) are measured in Hertz. Proton (^{1}H) NMR multiplicities are shown as s (singlet), d (doublet), t (triplet), q (quartet), m (multiplet), dd (double doublet), dt (double triplet), etc. HMBC, HSQC and DEPT were employed to aid with accurate assignments. Infrared spectra were recorded on a Perkin Elmer Spectrum 100 FTIR spectrometer (ATR mode). High and low resolution mass spectrometry of organic molecules was provided by the EPSRC Mass Spectrometry facility at Swansea using an LTQ Orbitrap XL.

Chemical Biology

All buffers were passed through a microfilter before use to remove particulates and the pH adjusted using 1 M HCl or 1 M NaOH. pH was measured using a Hanna Instruments pH 210 electronic pH meter. When spin filtration devices are mentioned VivaSpin (GE Healthcare) 10,000 Da weight cut off devices were employed. For small scale centrifugation Eppendorf 5415 R and VWR Galaxy 14D microcentrifuges were employed. An Eppendorf Thermomixer Comfort heating block was used for temperature and agitation-controlled experiments.

UV-Vis spectroscopy

Protein concentrations were determined photometrically using a ThermoScientific Nanodrop 2000C, and UV-Vis spectra were obtained using a Varian Cary 100 Bio UV-Visible spectrophotometer operating at 21 °C.

SDS-PAGE gels

Non-reducing 12% acrylamide gels were made using standard procedures. A 4% stacking gel was utilised. Samples (15 µM) were mixed 1:5 with a 5X R-250 Dye SDS-loading

buffer, heated for 3 minutes at 75 °C and loaded onto the gel with a total volume of 5 µL. Samples were run at constant current (30 mA) for 40 minutes in 1 x SDS running buffer and stained with Coomasie.

Protein LCMS

All proteins were prepared for analysis by repeated diafiltration into ammonium acetate buffer (50 mM ammonium acetate, pH 6.9) using VivaSpin sample concentrators (GE Healthcare, 10000 MWCO) to a concentration of 2 µM. Samples were submitted to the UCL Chemistry Mass Spectrometry Facility at the Chemistry Department, UCL for analysis on the Agilent 6510 QTOF LC-MS system (Agilent, UK). 10 µL of each sample was injected onto a PLRP-S, 1000A, 8 mM, 150 mm x 2.1 mm column, which was maintained at 60 °C. Flow rate was set at 0.600 ml/min. Solvent A is H_2O (0.1% formic acid), solvent B is MeCN (0.1% formic acid), and separation was achieved using a gradient elution. The column effluent was continuously electrosprayed into the capillary ESI source of the Agilent 6510 QTOF mass spectrometer and ESI mass spectra were acquired in positive electrospray ionisation (ESI) mode using the m/z range 1000 – 8000 in profile mode. The raw data was converted to zero charge mass spectra using maximum entropy deconvolution algorithms using MassHunter software (version B.07.00).

Fabrication of TIPS Microparticles

TIPS microparticles intended for click chemistry were prepared by blending together two polymers: poly(D, L-lactide-co-glycolide) (PLGA; PURASORB PDLG 7507 75/25 DL-lactide/glycolide copolymer; inherent viscosity 0.75 dl/g; Corbion, Netherlands) and PLGA azide-capped (PLGA-N_3; Mw ~95K; Nanosoft Polymers, USA). Each polymer was dissolved separately in dimethyl carbonate (Sigma Aldrich, Dorset, UK) using magnetic stirring to produce a 5 wt% polymer solution before specific volumes were mixed together to produce solutions with different v/v ratios of PLGA:PLGA-N_3 (0:100, 25:75, 50:50, 75:25, 100:0). The polymer solutions were mixed together by placing on a vortex for 10 seconds. The polymer solution was immediately fed into a Nisco Encapsulator Unit (Nisco Engineering, Zurich, Switzerland; Frequency: 2.75 kHz, Amplitude: 70%) by a syringe pump (Harvard Apparatus, Kent, UK), at a constant flow rate of 2 mL/min. The polymer droplets were formed using a 150 µm sapphire nozzle and collected in a liquid nitrogen quenching bath. Frozen solvent was removed from the

polymer droplets by lyophilisation for 48 h. The dried TIPS microparticles were stored in a desiccator at room temperature in rubber stoppered glass vials under vacuum.

Imaging Click-TIPS microparticles

Fluorescence microscopy was used to detect azide-capped PLGA on the surface of TIPS microparticles via staining with an azadibenzocyclooctyne-tetramethyl rhodamine (TAMRA) derivative. TIPS microparticles containing different ratios of PLGA and PLGA-N_3 (0:100, 25:75, 50:50, 75:25, 100:0) were transferred to 0.5 ml polypropylene microfuge tubes. 30µl of dibenzocyclooctyne-PEG4-Fluor 545 (20 mM stock solution in degassed DMSO diluted 1:200 (v/v) in PBS) was added to each sample and mixed. The samples were incubated for 18 hours in the dark at 37°C. The excess stain was removed and the sample washed four times with 500µl dH_2O. The microparticles were transferred onto carbon sticky tabs and imaged using a stereo microscope (Leica MZ10 F) under 545 nm excitation and 610 nm emission. To enable comparison of the fluorescence signal between different sample groups the auto exposure was disabled and imaging parameters were set using 0% azide (100% PLGA) microparticle before acquiring all images using the same camera settings.

Scanning electron microscopy was used to assess the size distribution of the microparticles and the surface ultrastructural features of TIPS microparticles composed of the different ratios of PURASORB PDLG 7507 and azide-capped PLGA. The microparticles were placed onto aluminium stubs and coated with gold for 60 seconds using a Quorum Technologies Q150R ES gold coater. The samples were imaged using a Hitachi S3400N scanning electron microscope. The diameter of 60 microparticles from the SEM images was measured using ImageJ software.

Anti-Fab HRP modified ELISA

Microparticles (2 mg 100:0 or 25:75 PLGA-N_3) were added to Costar® Spin-X® Centrifuge Tube Filters in duplicates. 190 µL of PBST (Phosphate buffer, pH = 7.4, 0.1% Tween 80) was added. Microparticles were then incubated with Herceptin™ Fab conjugate **2** (10 µL, 5 µM, concentration in solution = 0.25 µM), native Herceptin™ Fab **1** (10 µL, 5 µM, concentration in solution = 0.25 µM) or PBST for 16 h, at 21 °C. After incubation, the microparticles were separated from the incubation solutions via

centrifugation (15 s) before immersion in 400 µL PBST. The process was repeated (10 ×). Non-specific binding sites on the microparticles were blocked with PBST buffer containing 1% w/v bovine serum albumin (PBST BSA) for 30 min at 21 °C, after which washing was repeated. 200 µL of anti-human IgG (Fab specific)−peroxidase conjugated antibody (Anti-Fab HRP) in PBST (1:50000 dilution) was added to the microparticles and incubated for 2 h at 21 °C. The microparticles were then washed 10 times in PBST *via* filter centrifugation. The filters with the microparticles were transferred to 2 mL containers and freshly prepared o-phenylenediamine dihydrochloride (OPDD) substrate solution was added (100 µL each) and incubated for 20 min at 21 °C with the exclusion of light. The filters with the microparticles were removed and stop solution (H_2SO_4, 1 M, 50 µL) was added. 100 µL of supernatant was transferred to a 96-well plate and the absorbance was read at 490 nm.

HER2 binding modified ELISA

Microparticles (1 mg 25:75 PLGA-N_3) were added to Costar® Spin-X® Centrifuge Tube Filters in triplicate and 90 µL of PBST (Phosphate buffer, pH = 7.4, 0.1% Tween 80) was added. Microparticles were incubated with Herceptin™ Fab conjugate **2** (5 µL, 5 µM, concentration in solution = 0.25 µM), native Herceptin™ Fab **1** (5 µL, 5 µM, concentration in solution = 0.25 µM) or PBST for 16 h, at 21 °C. After incubation, the microparticles were separated from the incubation solutions via centrifugation (15 s) before immersion in 400 µL PBST. The process was repeated (10 ×). Non-specific binding sites on the microparticle were blocked with PBST buffer containing 1% w/v bovine serum albumin (PBST BSA) for 30 min at 21 °C, after which washing was repeated. 100 µL of 4 nM Biotin-conjugated HER2 (MABSol® Biotinylated Human HER2) was incubated with appropriate microparticle controls (HER2+ and PBS controls) for 1 h at 21 °C. For HER2- control, no biotin-conjugated HER2 was added. The microparticles were then washed 10 times in PBST *via* filter centrifugation. 100 µL of 4 nM of HRP-conjugated streptavidin (Thermo Scientific™) was added to the microparticles and incubated for 1 h at 21 °C. The microparticles were then washed 10 times in PBST *via* filter centrifugation. The filters with the microparticles were transferred to 2 mL containers and freshly prepared OPDD substrate solution was added (100 µL each) and incubated for 20 min at 21 °C with the exclusion of light. The filters with the microparticles were removed and stop solution (H_2SO_4, 1 M, 50 µL) was added.

100 µL of supernatant was transferred to a 96-well plate and the absorbance was read at 490 nm.

Synthesis of compounds

Di-*tert*-butyl 1-methylhydrazine-1,2-dicarboxylate[1]

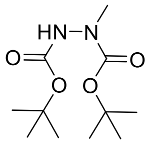

To a stirring solution of *N*-methylhydrazine (157 mg, 3.42 mmol) in *i*-PrOH (4.3 mL) was added dropwise di-*tert*-butyl dicarbonate (1.6 g, 7.5 mmol, pre-dissolved in CH_2Cl_2 (3.4 mL)) over 20 min. The mixture was then stirred for 16 h at 21 °C. After this time, solvent was removed *in vacuo* and purification by flash column chromatography (20% Et_2O/petrol) yielded di-*tert*-butyl 1-methylhydrazine-1,2-dicarboxylate (407 mg, 2.05 mmol, 60%) as a white solid: m.p. 58–62 °C (*lit m.p.* 54–56 °C). ^{1}H NMR (600 MHz, $CDCl_3$, rotamers) δ 6.55-6.10 (br s, 1H), 3.11 (s, 3H), 1.47 (s, 18H); ^{13}C NMR (150 MHz, $CDCl_3$, rotamers) *δ* 155.9 (C), 81.3 (C), 37.5 (CH_3), 28.3 (CH_3); IR (solid) 3316, 2978, 2932, 1701 cm^{-1}. Representative spectra from *Org. Biomol. Chem.*, 2018, **16**, 1359–1366 provided below:

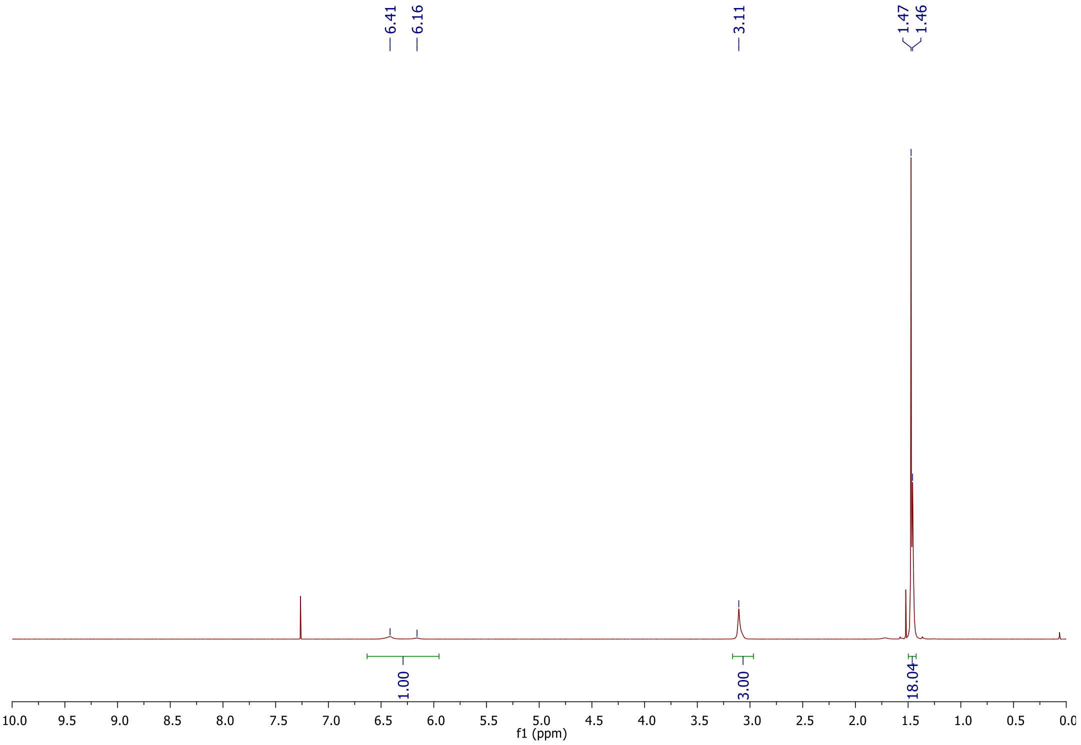
6.41
6.16
3.11
1.47
1.46
1.00
3.00
18.04
10.0 9.5 9.0 8.5 8.0 7.5 7.0 6.5 6.0 5.5 5.0 4.5 4.0 3.5 3.0 2.5 2.0 1.5 1.0 0.5 0.0
f1 (ppm)

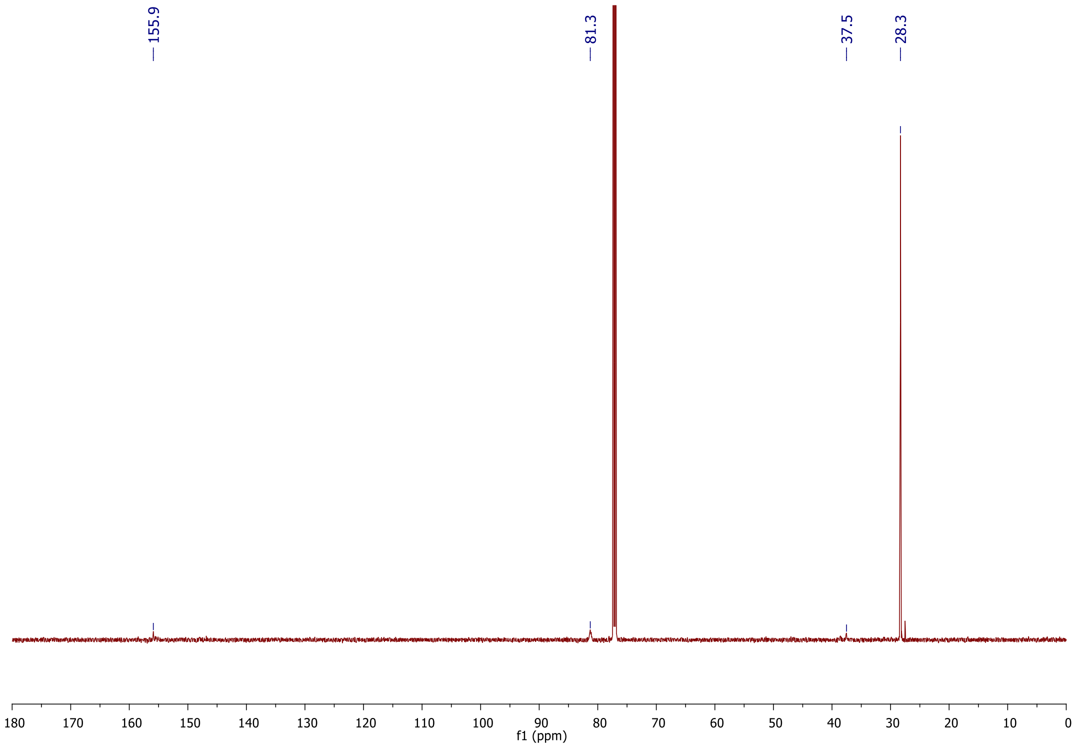
155.9
81.3
37.5
28.3
180 170 160 150 140 130 120 110 100 90 80 70 60 50 40 30 20 10 0
f1 (ppm)

Di-*tert*-butyl 1-(3-(tert-butoxy)-3-oxopropyl)-2-methylhydrazine-1,2-dicarboxylate[1]

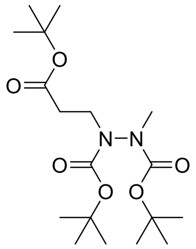

To a solution of di-*tert*-butyl 1-methylhydrazine-1,2-dicarboxylate (3.00 g, 12.2 mmol) in *t*-BuOH (5 mL), was added 10% NaOH (0.5 mL) and the reaction mixture stirred at 21 °C for 10 min. After this, *tert*-butyl acrylate (5.31 mL, 36.6 mmol) was added to the solution and the reaction mixture was heated at 60 °C for 24 h. Following this, the solvent was removed *in vacuo* and the crude residue was dissolved in EtOAc (150 mL) and washed with water (3 × 50 mL). The organic layer was then dried ($MgSO_4$) and concentrated *in vacuo*. Purification of the residue by flash column chromatography (0% to 20% EtOAc/petrol) afforded di-*tert*-butyl-1-(3-(*tert*-butoxy)-3-oxopropyl)-2-methylhydrazine-1,2-dicarboxylate (2.24 g, 5.98 mmol, 49%) as a clear oil. ^{1}H NMR (600 MHz, $CDCl_3$, rotamers) δ 3.85–3.52 (m, 2H), 3.06–2.99 (m, 3H), 2.51 (t, *J* = 7.2 Hz, 2H), 1.48–1.43 (m, 27H). ^{13}C NMR (150 MHz, $CDCl_3$, rotamers) δ 171.0 (C), 155.4 (C), 154.4 (C), 81.0 (C), 44.6 (CH_3), 36.6 (CH_2), 34.1 (CH_2), 28.3 (CH_3). IR (thin film) 2976, 2933, 1709 cm^{-1}. LRMS (ESI) 375 (100, $[M+H]^+$), 319 (30, $[M-C_4H_9+2H]^+$. HRMS (ESI) calcd for $C_{18}H_{35}N_2O_6$ $[M+H]^+$ 376.2524; observed 376.2516. Representative spectra from *Org. Biomol. Chem.*, 2018, **16**, 1359–1366 provided below:

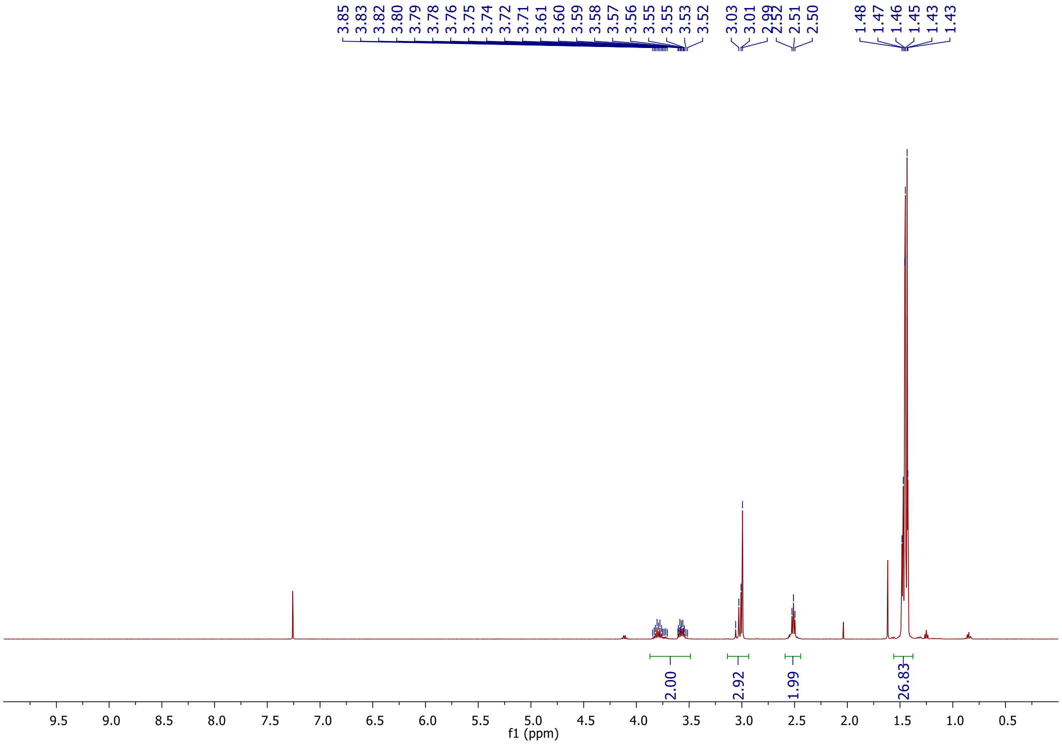
3.85 3.83 3.82 3.80 3.79 3.78 3.76 3.75 3.74 3.72 3.71 3.61 3.60 3.59 3.58 3.57 3.56 3.55 3.55 3.53 3.52
3.03 3.01 2.99 2.52 2.51 2.50
1.48 1.47 1.46 1.45 1.43 1.43
2.00
2.92
1.99
26.83
9.5 9.0 8.5 8.0 7.5 7.0 6.5 6.0 5.5 5.0 4.5 4.0 3.5 3.0 2.5 2.0 1.5 1.0 0.5
f1 (ppm)
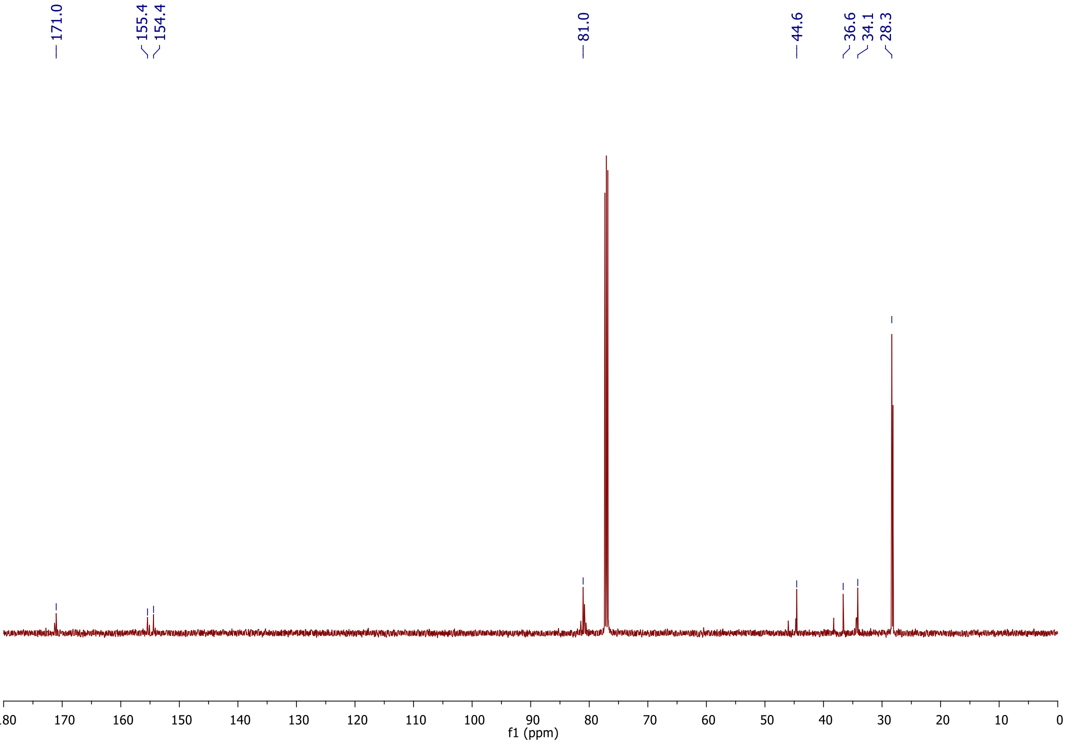
171.0
155.4
154.4
81.0
44.6
36.6
34.1
28.3
180 170 160 150 140 130 120 110 100 90 80 70 60 50 40 30 20 10 0
f1 (ppm)

3-(4,5-Dibromo-2-methyl-3,6-dioxo-3,6-dihydropyridazin-1(2*H*)-yl) propanoic acid[1]

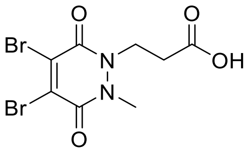

Dibromomaleic acid (4.93 g, 17.9 mmol) was dissolved in AcOH (25 mL) and heated under reflux for 30 min. To this solution, was added di-*tert*-butyl-1-(3-(*tert*-butoxy)-3-oxopropyl)-2-methylhydrazine-1,2-dicarboxylate (5.60 g, 14.9 mmol) and the reaction heated under reflux for a further 4 h. After this time, the reaction mixture was then concentrated *in vacuo* with toluene co-evaporation (3 × 30 mL, as an azeotrope) and the crude residue purified by flash column chromatography (50% to 100% EtOAc/petrol (1% AcOH)) to afford 3-(4,5-dibromo-2-methyl-3,6-dioxo-3,6-dihydropyridazin-1(2*H*)-yl) propanoic acid (3.41 g, 9.57 mmol, 64%) as a yellow solid. m.p. 140–144 °C ^{1}H NMR (600 MHz, DMSO-d_6) δ 4.28 (t, *J* = 7.3 Hz, 2H), 3.56 (s, 3H), 2.63 (t, *J* = 7.3 Hz, 2H). ^{13}C NMR (150 MHz, DMSO-d_6) δ 171.9 (C), 152.7 (C), 152.4 (C), 135.3 (C), 135.0 (C), 43.1 (CH_3), 34.7 (CH_2), 31.7 (CH_2). IR (solid) 3044, 1725, 1606, 1570 cm^{-1}. LRMS (ESI) 359 (50, $[M^{81}Br^{81}Br+H]^+$) 357 (100, $[M^{79}Br^{81}Br+H]^+$), 355 (50, $[M^{79}Br^{79}Br+H]^+$). HRMS (ESI) calcd for $C_8H_9Br_2N_2O_4$ $[M^{79}Br^{81}Br+H]^+$ 358.8883; observed 358.8882. Representative spectra from *Org. Biomol. Chem.*, 2018, **16**, 1359–1366 provided below:

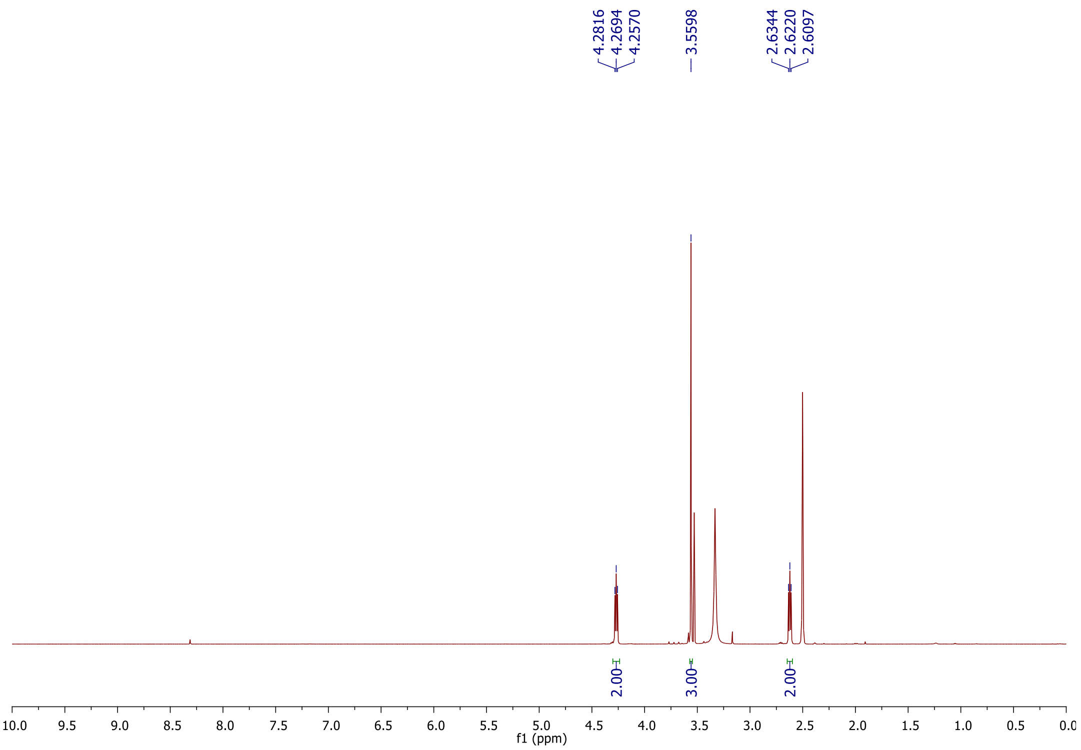
4.2816
4.2694
4.2570
3.5598
2.6344
2.6220
2.6097
2.00
3.00
2.00
10.0
9.5
9.0
8.5
8.0
7.5
7.0
6.5
6.0
5.5
5.0
4.5
4.0
3.5
3.0
2.5
2.0
1.5
1.0
0.5
0.0
f1 (ppm)

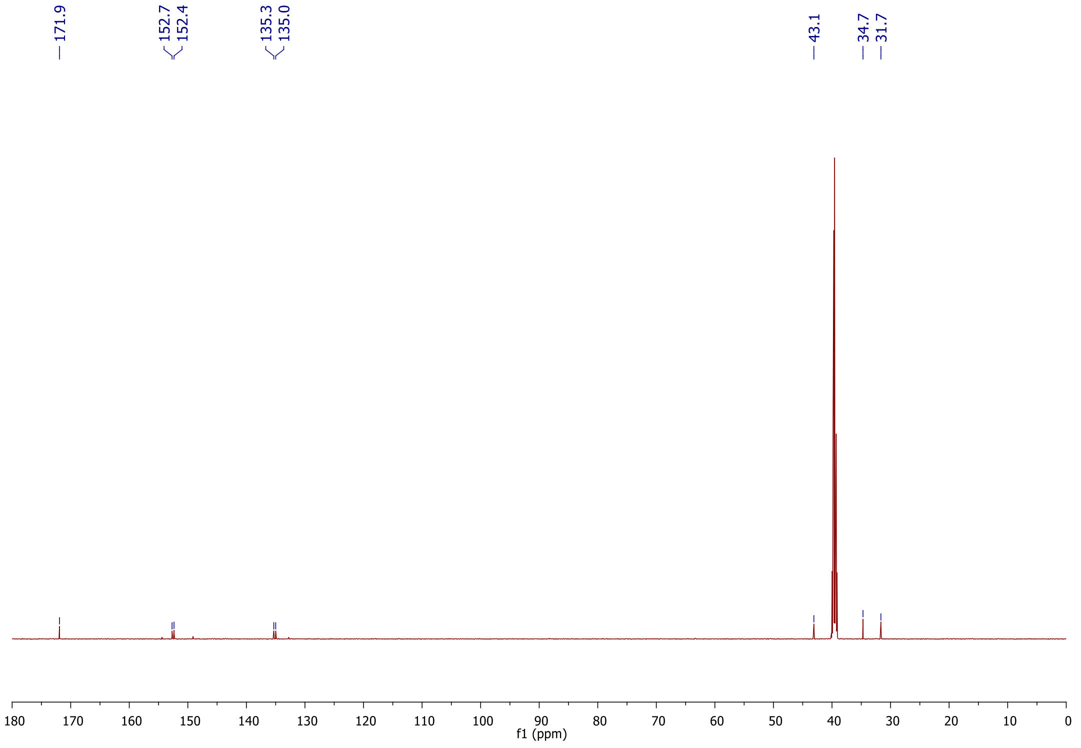
171.9
152.7
152.4
135.3
135.0
43.1
34.7
31.7
180
170
160
150
140
130
120
110
100
90
80
70
60
50
40
30
20
10
0
f1 (ppm)

2,5-Dioxopyrrolidin-1-yl 3-(4,5-dibromo-2-methyl-3,6-dioxo-3,6-dihydropyridazin-1(2*H*)-yl) propanoate[1]

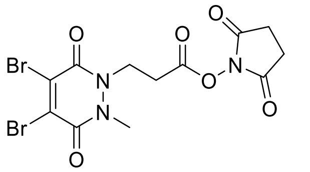

A solution of 3-(4,5-dibromo-2-methyl-3,6-dioxo-3,6-dihydropyridazin-1(2*H*)-yl) propanoic acid (750 mg, 2.13 mmol) in THF (10 mL) was cooled to 0 °C and was added N,N'-dicyclohexylcarbodiimide (480 mg, 2.34 mmol). The homogenous solution was then stirred at 0 °C for 30 min. After this time, was added *N*-hydroxysuccinimide (89 mg, 0.78 mmol) and the reaction stirred at 21 °C for a further 16 h. The newly formed heterogenous mixture was then filtered and the filtrate concentrated in *vacuo*. Purification of the crude residue by flash column chromatography (20% to neat EtOAc/petrol) afforded 2,5-dioxopyrrolidin-1-yl 3-(4,5-dibromo-2-methyl-3,6-dioxo-3,6-dihydropyridazin-1(2*H*)-yl) propanoate (511 mg, 1.13 mmol, 53%) as a white solid. m.p. 100–104 °C. ^{1}H NMR (600 MHz, $CDCl_3$) δ 4.48 (t, J = 6.9 Hz, 2H), 3.68 (s, 3H), 3.11 (t, J = 6.9 Hz, 2H), 2.85 (s, 4H). ^{13}C NMR (150 MHz, $CDCl_3$) δ 168.7 (C), 166.0 (C), 153.3 (C), 153.1 (C), 136.9 (C), 135.3 (C), 43.0 (CH_2), 35.3 (CH_3), 29.1 (CH_2), 25.7 (CH_2). IR (solid) 2992, 1814, 1782, 1735, 1634, 1576 cm^{-1}. LRMS (ESI) 358 (50, [M^{81}Br^{81}Br+H-succinimide]$^+$), 356 (100, [M^{81}Br^{79}Br+H-succinimide]$^+$), 354 (50, [M^{79}Br^{79}Br+H-succinimide]$^+$). Unable to obtain HRMS data due to decomposition of the NHS ester under mass spec conditions. Representative spectra from *Org. Biomol. Chem.*, 2018, **16**, 1359–1366 provided below:

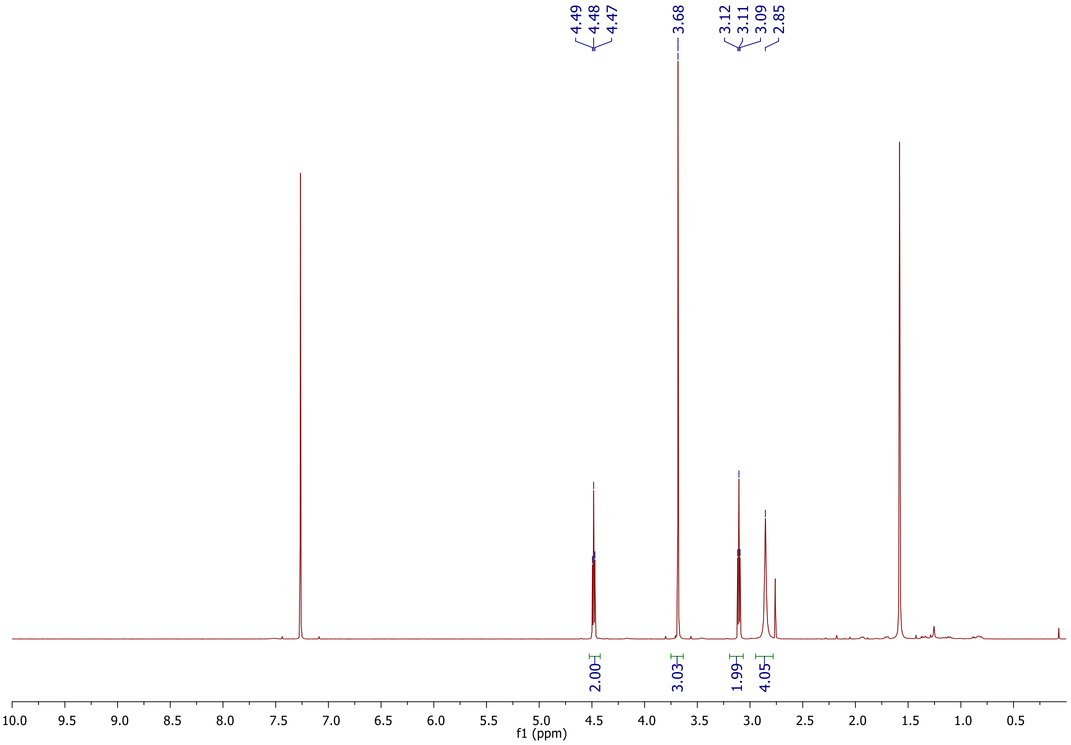
4.49
4.48
4.47
3.68
3.12
3.11
3.09
2.85
2.00
3.03
1.99
4.05
10.0 9.5 9.0 8.5 8.0 7.5 7.0 6.5 6.0 5.5 5.0 4.5 4.0 3.5 3.0 2.5 2.0 1.5 1.0 0.5
f1 (ppm)

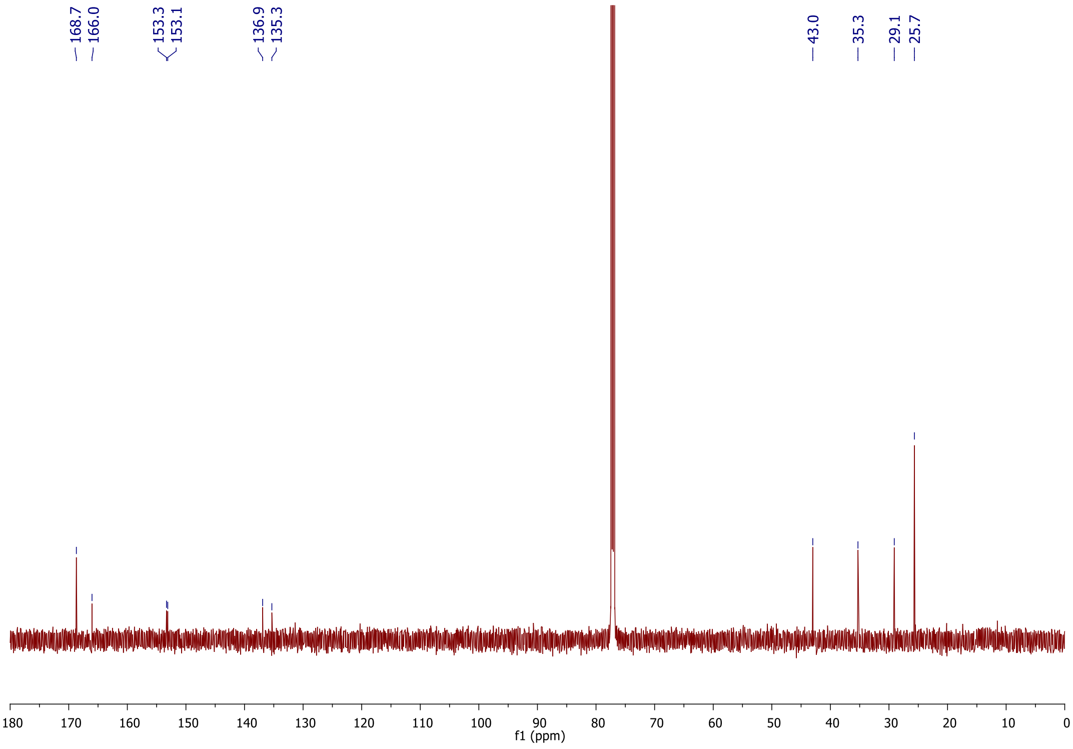
168.7
166.0
153.3
153.1
136.9
135.3
43.0
35.3
29.1
25.7
180 170 160 150 140 130 120 110 100 90 80 70 60 50 40 30 20 10 0
f1 (ppm)

((1*R*,8*S*,9*S*)-Bicyclo[6.1.0]non-4-yn-9-yl)methyl (2-(2-(2-(3-(4,5-dibromo-2-methyl-3,6-dioxo-3,6-dihydropyridazin-1(2*H*)-yl)propanamido)ethoxy)ethoxy)ethyl) carbamate[1] **4**

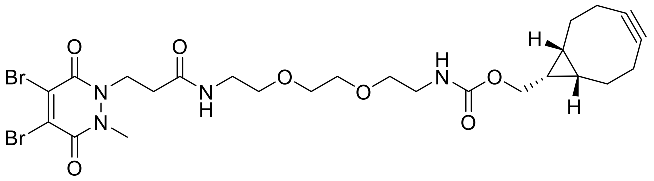

To a solution of 2,5-dioxopyrrolidin-1-yl 3-(2-bromo-2-methyl-3,6-dioxo-3,6-dihydropyridazin-1(2*H*)yl) propanoate (132 mg, 0.200 mmol, pre-dissolved in MeCN (10 mL)), was added *N*-[(1*R*,8*S*,9*S*)-bicyclo[6.1.0]non-4-yn-9-ylmethyloxycarbonyl]-1,8-diamino-3,6-dioxaoctane (71 mg, 0.220 mmol) and the reaction mixture was stirred at 21 °C for 16 h. After this time, the reaction mixture was concentrated *in vacuo* and the crude residue dissolved in $CHCl_3$ (10 mL) and washed with water (2 × 5 mL) and saturated aq. K_2CO_3 (10 mL). The organic layer was then dried ($MgSO_4$) and concentrated *in vacuo*. Purification of the crude residue by flash column chromatography (0% to 10% MeOH/EtOAc) afforded ((1*R*,8*S*,9*S*)-Bicyclo[6.1.0]non-4-yn-9-yl)methyl (2-(2-(2-(3-(4,5-dibromo-2-methyl-3,6-dioxo-3,6-dihydropyridazin-1(2*H*)-yl)propanamido)ethoxy)ethoxy)ethyl) carbamate **4** (105 mg, 0.160 mmol, 72%) as a yellow oil: ^{1}H NMR (600 MHz, $CDCl_3$, rotamers) δ 7.84 (s, 0.5H), 6.34 (s, 0.5H), 5.82 (s, 0.5H), 5.29 (s, 0.5H), 4.44 (t, *J* = 6.6 Hz, 2H), 4.14–4.12 (m, 2H), 3.73–3.71 (m, 3H), 3.60–3.38 (m, 12H), 2.62 (t, *J* = 6.6 Hz, 2H), 2.29-2.22 (m, 6H), 1.61–1.57 (m, 2H), 1.39–1.24 (m, 1H), 0.96–0.94 (m, 2H); ^{13}C NMR (150 MHz, $CDCl_3$, rotamers) δ 169.1 (C), 156.9 (C), 153.1 (C), 153.0 (C), 136.4 (C), 135.5 (C), 98.9 (C), 70.4 (CH_2), 70.3 (CH_2), 69.7 (CH_2), 63.0 (CH_2), 44.6 (CH_2), 40.8 (CH_2), 39.5 (CH_2), 35.1 (CH_3), 34.1 (CH_2), 29.3 (CH_2), 29.2 (CH_2), 21.6 (CH_2), 20.2 (CH_2), 17.9 (CH), 14.3 (CH); IR (thin film) 3329, 2920, 2858, 1708, 1630, 1572, 1534 cm^{-1}; LRMS (ESI), 687 (50, $[M^{81}Br^{81}Br+Na]^+$) 685 (100, $[M^{79}Br^{81}Br+Na]^+$), 683 (50, $[M^{79}Br^{79}Br+Na]^+$), 663 (60, $[M^{79}Br^{81}Br+H]^+$); HRMS (ESI) calcd for $C_{25}H_{35}Br_2N_4O_7$ $[M^{79}Br^{81}Br+H]^+$ 663.0847; observed 663.0846. Representative spectra from *Org. Biomol. Chem.*, 2018, **16**, 1359–1366 provided below:

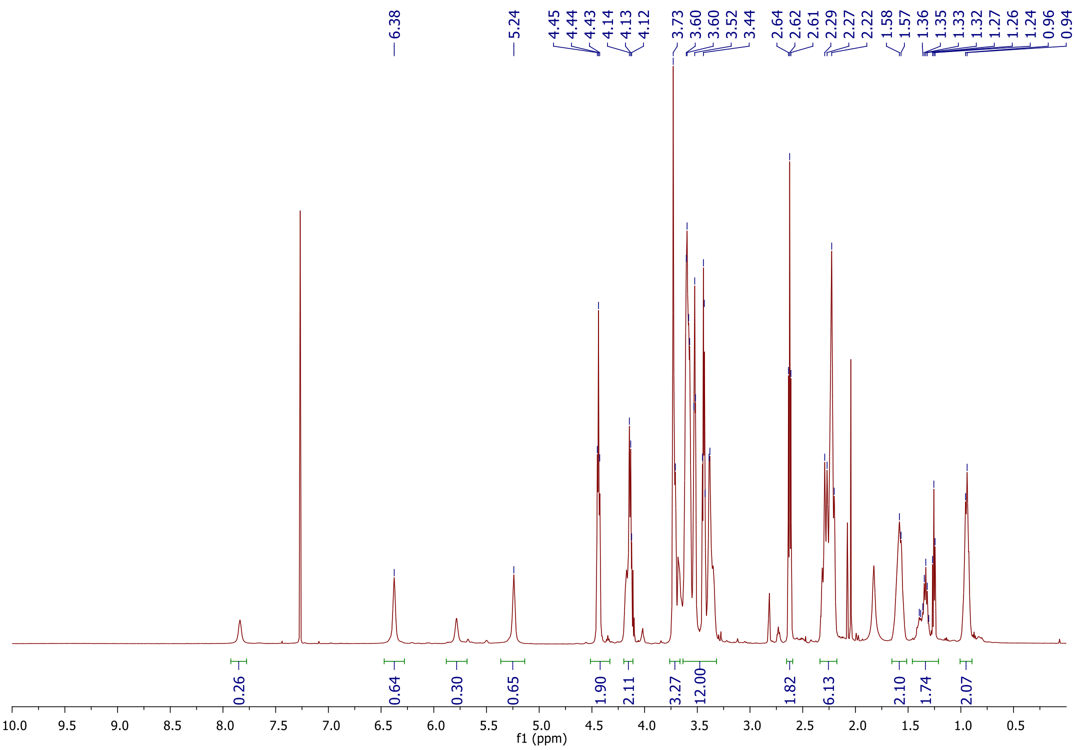

6.38
5.24
4.45
4.44
4.43
4.14
4.13
4.12
3.73
3.60
3.60
3.52
3.44
2.64
2.62
2.61
2.29
2.27
2.22
1.58
1.57
1.36
1.35
1.33
1.32
1.27
1.26
1.24
0.96
0.94
0.26
0.64
0.30
0.65
1.90
2.11
3.27
12.00
1.82
6.13
2.10
1.74
2.07
10.0 9.5 9.0 8.5 8.0 7.5 7.0 6.5 6.0 5.5 5.0 4.5 4.0 3.5 3.0 2.5 2.0 1.5 1.0 0.5
f1 (ppm)

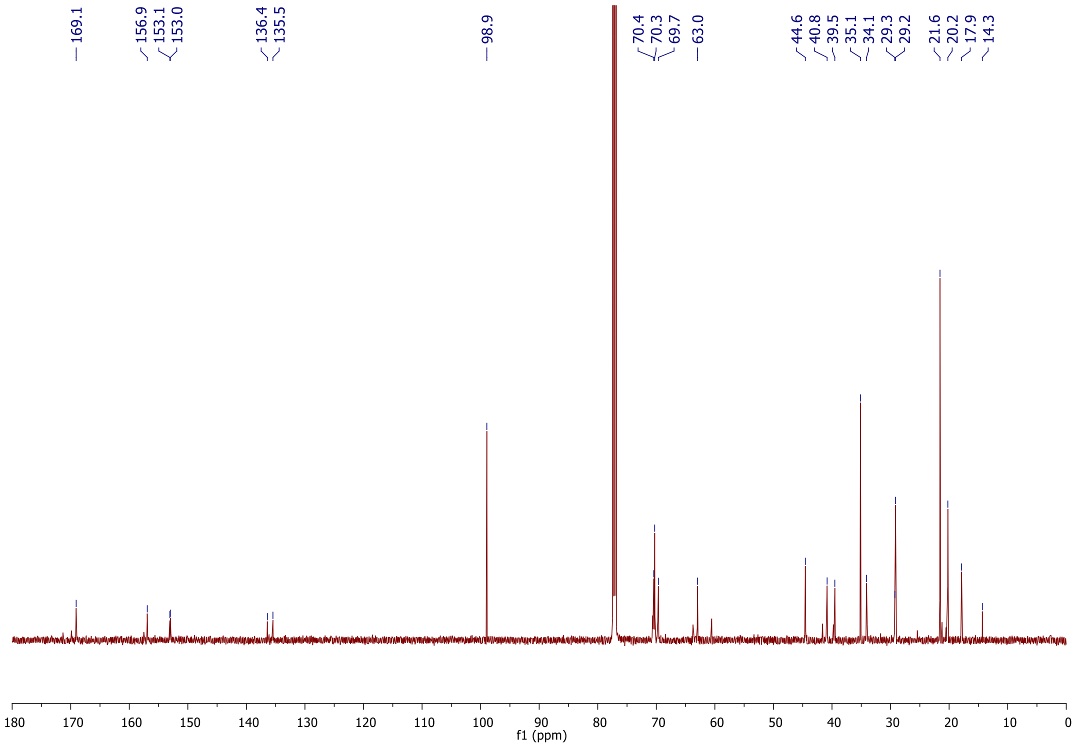

169.1
156.9
153.1
153.0
136.4
135.5
98.9
70.4
70.3
69.7
63.0
44.6
40.8
39.5
35.1
34.1
29.3
29.2
21.6
20.2
17.9
14.3
180 170 160 150 140 130 120 110 100 90 80 70 60 50 40 30 20 10 0
f1 (ppm)

Chemical Biology

Herceptin™ Fab 1

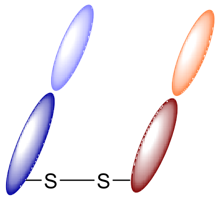

Immobilized pepsin (0.6 mL) was washed with acetate buffer (4 × 0.3 mL, 20 mM sodium acetate trihydrate, pH 3.1). Herceptin™ (19.5 mg, 3 mL, 6.55 mg·mL^{-1} in acetate buffer) was added and the mixture incubated for 5 h under constant agitation (1100 rpm) at 37 °C. The digest solution was separated from the resin beads, which were washed with digest buffer (3 × 0.4 mL). The washings were combined to yield Herceptin™ F(ab')$_2$, confirmed by SDS-PAGE. After this, immobilised papain (2 mL, 0.25 mg·mL^{-1}) was incubated in buffer (10 mM DTT in digest buffer) at 37 °C with constant agitation (1100 rpm) for 1 h. The papain resin was subsequently filtered and washed with digest buffer (4 × 0.4 mL), and Herceptin™ F(ab')$_2$ was added to the beads. The mixture was incubated at 37 °C with constant agitation (1100 rpm) for 16 h, before being separated from the digest *via* spin filtration and washed with BBS (3 × 0.4 mL, 25 mM sodium borate, 25 mM NaCl, 0.5 mM EDTA). The digest and the washes were combined and buffer swapped for BBS using spin filtration columns (10,000 MWCO), and the volume was adjusted to 0.5 mL. Herceptin™ Fab **1** (7.2 mg, 82% yield) was confirmed using SDS-PAGE and LC-MS. Yield of **1** was determined by UV/Vis spectroscopy (ε_{280} = 68,590 M^{-1}·cm^{-1}). Expected mass: 47637 Da. Observed mass: 47640 Da.

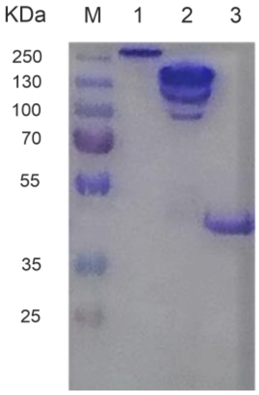

Figure 1. SDS-PAGE gel for Herceptin™ digestion**: M)** Molecular weight marker. **1)** Herceptin™. **2)** Herceptin™ F(ab')$_2$. **3)** Herceptin™ Fab **1**.

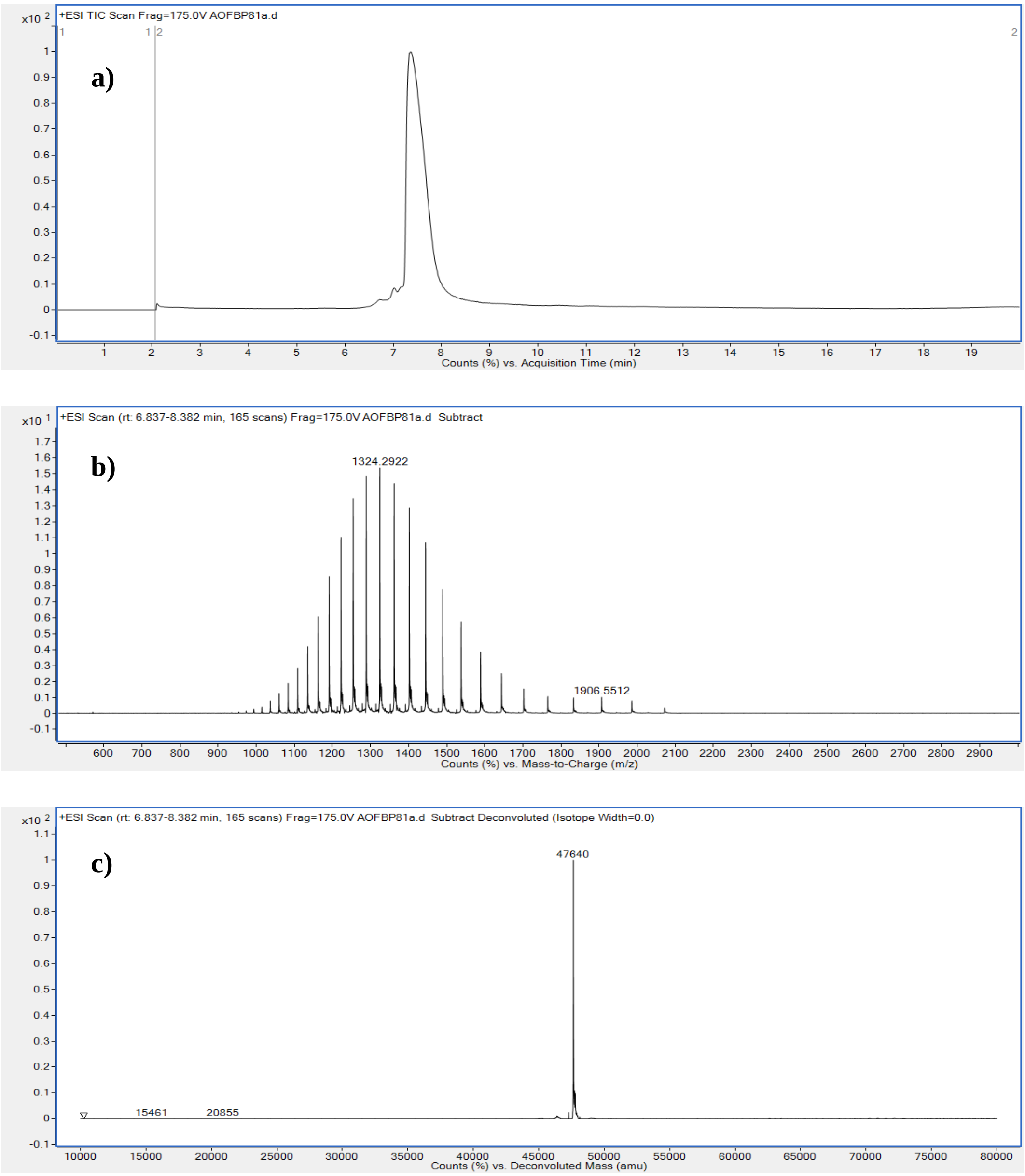

Figure 2. (a) TIC, **(b)** non-deconvoluted LC-MS trace and **(c)** deconvoluted MS data for Herceptin™ Fab fragment **1**.

Herceptin™ Fab modification and "click" test

Herceptin™ Fab conjugate **2**

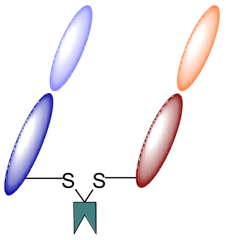

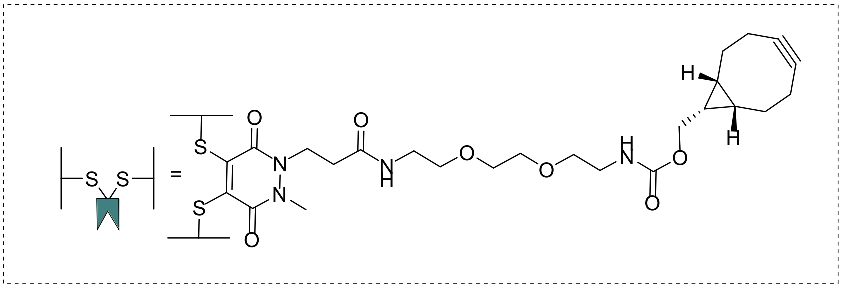

To a solution of Herceptin™ Fab **1** (50 µL, 20 µM, 1 eq.) in BBS (25 mm sodium borate, 25 mm NaCl, 5 mm EDTA, pH 8.0 + 3% DMSO) was added PD **4** (0.5 µL, 20 mM in DMSO, 10 eq.), followed by TCEP (0.5 µL, 20 mM in H_2O, 10 eq.) and the reaction mixture incubated at 21 °C for 16 h. The excess reagents were then removed by repeated diafiltration into fresh buffer using VivaSpin sample concentrators (GE Healthcare, 10,000 MWCO). Following this, analysis by SDS-PAGE, LCMS and UV-Vis revealed complete conversion to Herceptin™ Fab conjugate **2**. Expected mass: 48140 Da. Observed mass: 48143 Da.

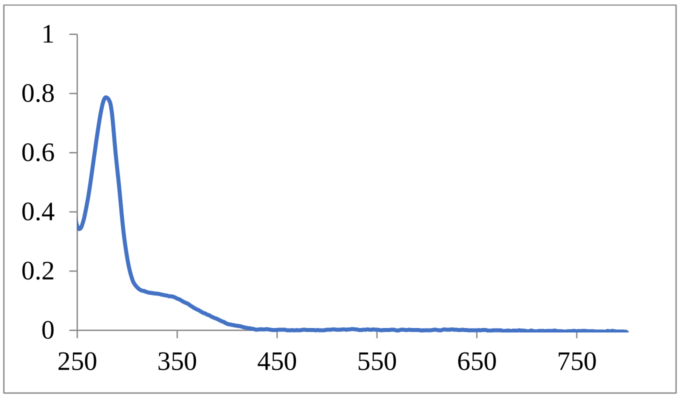

Figure 3. UV-Vis data for Herceptin™ Fab conjugate **2**, Pyridazinedione to antibody ratio ≈ 1.

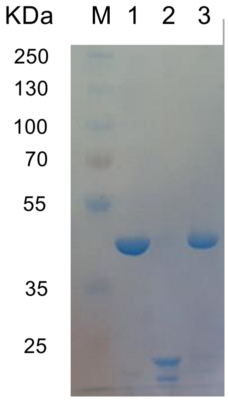

Figure 4. SDS-PAGE gel for successful formation of Herceptin™ Fab conjugate **2: M)** Molecular weight marker. **1)** Herceptin™ Fab **1**. **2)** Reduced Herceptin™ Fab (10 eq. TCEP). **3)** Herceptin™ Fab conjugate **2**.

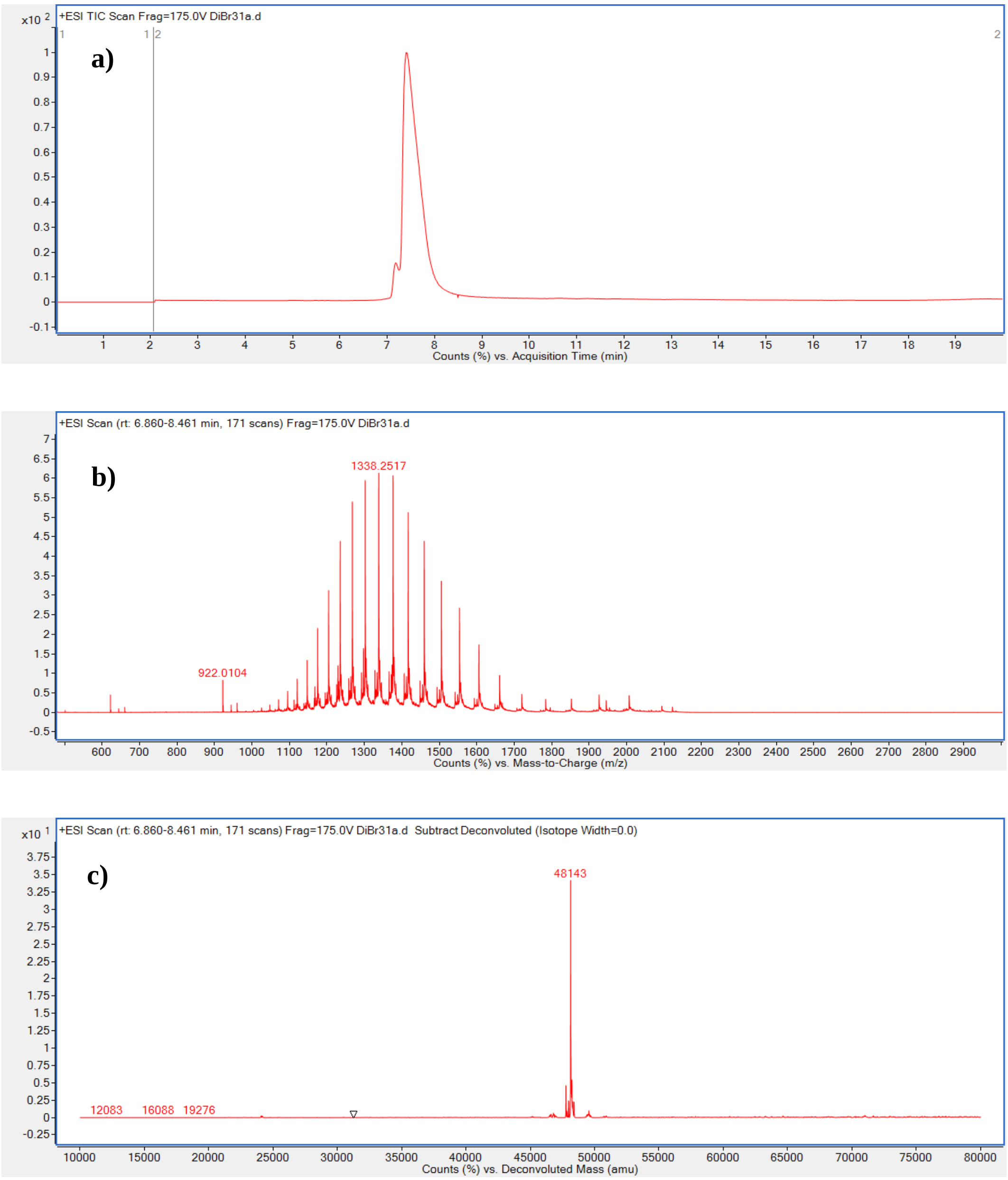

Figure 5. **(a)** TIC, **(b)** non-deconvoluted LCMS trace, **(c)** deconvoluted MS data for Herceptin™ conjugate **2**.

Herceptin™ Fab Alexafluor **3**

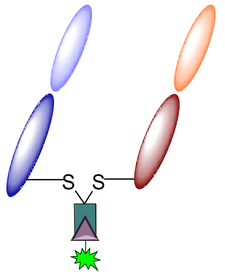

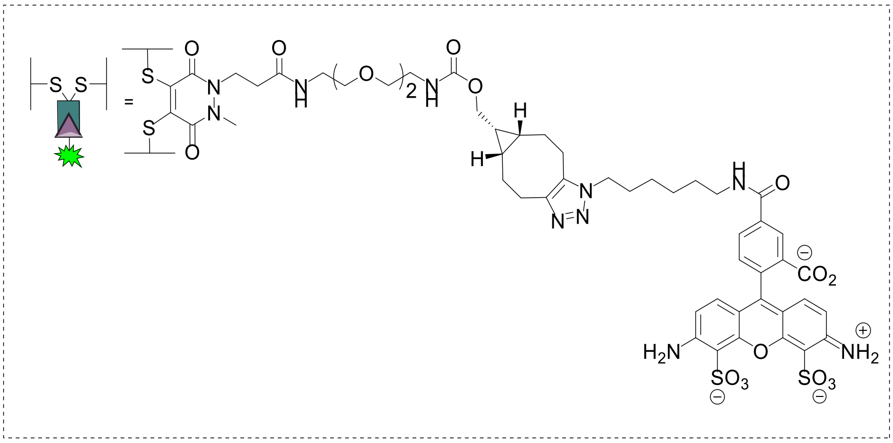

To a solution of Herceptin™ Fab conjugate **2** (50 µL, 20 µM, 1 eq.) in PBS (pH = 7.4) was added Alexafluor®-488-N_3 (0.2 µL, 20 mM in DMSO, 4 eq.) and the reaction mixture incubated at 21 °C for 2 h. The excess reagents were then removed by repeated diafiltration into fresh buffer using VivaSpin sample concentrators (GE Healthcare, 10,000 MWCO). Successful conjugation to Herceptin™ Fab Alexafluor **3** was confirmed by SDS-PAGE, and LC-MS. Expected mass: 48797 Da. Observed mass: 48800 Da.

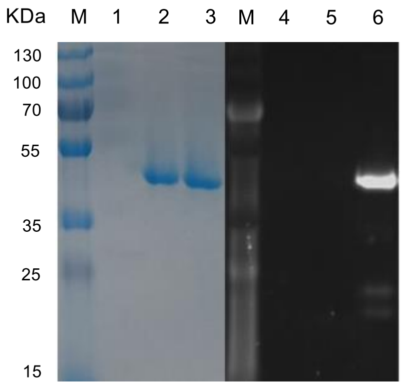

Figure 6. SDS-PAGE gel for successful formation of Herceptin™ Fab Alexafluor **3:** **M)** Molecular weight marker. **1)** Empty. **2)** Herceptin™ Fab conjugate **2**.

3) Herceptin™ Fab Alexafluor **3. 4)** Empty. **5)** Herceptin™ Fab conjugate **2** under UV light. **6)** Herceptin™ Fab Alexafluor **3** under UV light.

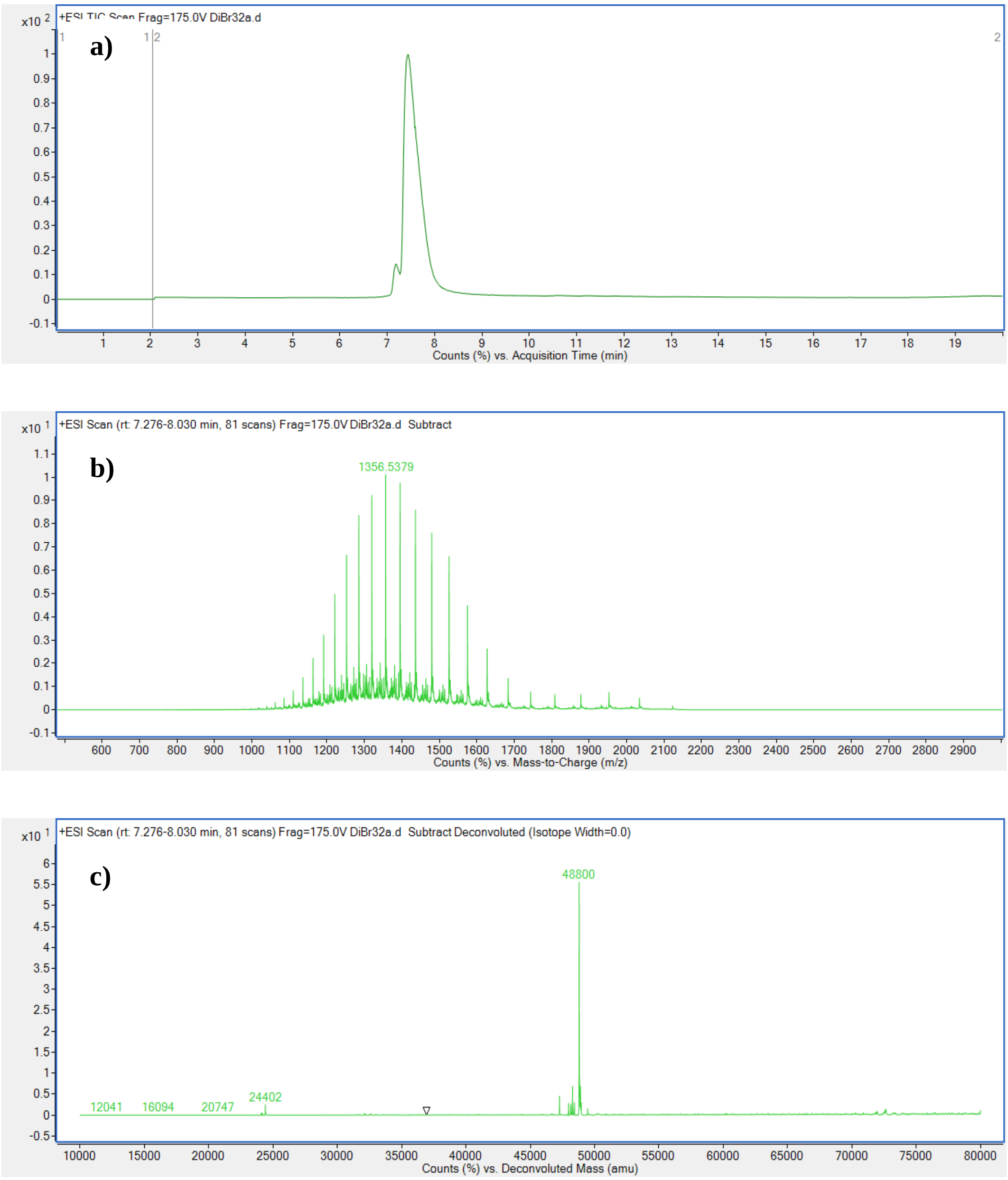

Figure 7. (a) TIC, **(b)** non-deconvoluted LCMS trace, **(c)** deconvoluted MS data for Herceptin™ Fab Alexafluor **3**.

TIPS microparticles characterisation

Scanning Electron Microscope (SEM) Imaging

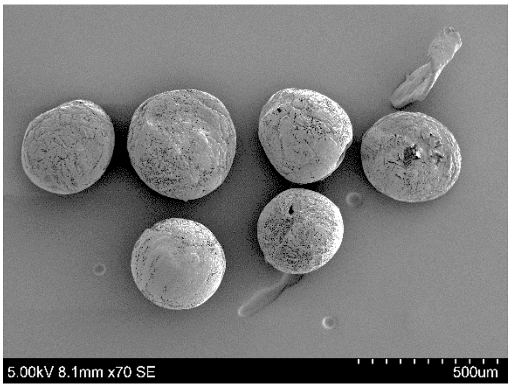

Figure 8. SEM image of TIPS microparticles (25:75 PLGA-N_3) .

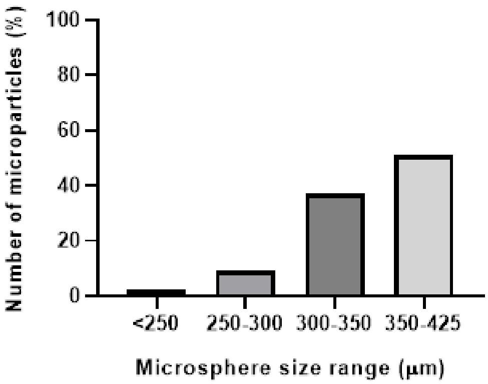

Figure 9. Size distribution of TIPS microparticles (25:75 PLGA-N_3) based on SEM images of 60 microparticles.

References

1 C. Bahou, D. A. Richards, A. Maruani, E. A. Love, F. Javaid, S. Caddick, J. R. Baker and V. Chudasama, *Org. Biomol. Chem.*, 2018, **16**, 1359–1366.
